# Editorial: The role of commensal microbiota in drug metabolism: friend or foe?

**DOI:** 10.3389/fmicb.2024.1364747

**Published:** 2024-01-30

**Authors:** Jing-Hua Wang, Dong-Woo Lim, Mukesh Kumar Yadav, Xiao Zheng

**Affiliations:** ^1^Institute of Bioscience and Integrative Medicine, College of Korean Medicine, Daejeon University, Daejeon, Republic of Korea; ^2^Department of Diagnostics, College of Korean Medicine, Dongguk University, Goyang, Republic of Korea; ^3^Department of Microbiology, Central University of Punjab, Bathinda, India; ^4^Laboratory of Metabolic Regulation and Drug Target Discovery, School of Pharmacy, China Pharmaceutical University, Nanjing, China

**Keywords:** drug metabolism, gut microbiome, metagenomics, precision medicine, personalized medicine, biotransformation, microbe-host co-metabolism

The commensal microbiome refers to the microorganisms widely inhabiting various parts of the host, primarily in the gastrointestinal tract (Dieterich et al., 2018). Understanding the interplay between drugs and the commensal microbiome is increasingly recognized as a critical aspect of pharmacology, pharmacokinetics, and personalized medicine (Wan and Zuo, 2022). Numerous researchers are aggressively exploring the potential to manipulate the gut microbiota to enhance drug efficacy, reduce toxicity, and develop more precise therapeutic interventions (Koppel et al., 2017). To further elucidate the underlying intricate mechanisms of bidirectional regulation between commensal microbiota and drugs, we hereby initiated the current Research Topic titled “*The role of commensal microbiota in drug metabolism: friend or foe?*”. Eventually, our topic collected six articles contributed by 30 authors from seven different countries, comprising two original research articles and four review articles focusing on specific drugs (anti-obesity drugs, antipsychotics, and opioids), artificial intelligence prediction, personalized medicine, and so forth.

It is widely accepted that drugs can interact with gut microbiota through various mechanisms and routes. Song et al. reported that lorcaserin (LS) and phentermine (PT), clinical anti-obesity drugs, can ameliorate gut microbiota dysbiosis, which in turn has a positive impact on obesity. Although causality is unidentified, gut microbiota together with LS and PT provided a synergistic effect on obesity. Overall, this study provides valuable insights into the potential new treatments for obesity that target gut microbiota, and further research in this area could lead to the development of new treatments for obesity (Song et al.). Moreover, Misera et al. conducted a narrative review that explores the relationship between psychotropic drugs and microbiota. They discussed how psychotropic drugs can have antimicrobial effects and how antibiotics can affect the mental phenotype and neurological function of the patients. The review also examined how alteration in the microbiota composition can lead to changes in the central nervous system. The authors have provided an overview of the current research on the topic and highlight the potential clinical significance of microbiota changes under the influence of psychotropic drugs. This informative review sheds light on the fascinating but complex relationship between drugs and microbiota and provides perceptions of how they can affect each other in surprising ways (Misera et al.). In addition, Kolli and Roy explored the fascinating connection between the gut microbiome/microbial metabolism and opioid-induced epigenetic changes. Their article has provided valuable insights into the fundamental significance of gut microbial metabolism over host epigenetic changes and behavior responses related to opioid usage. By gathering the available literature and data to date, these authors have broadened our understanding of the potential mechanism and role of the gut microbiome underlying the opioid pandemic and offered new perspectives for addressing this public health crisis (Kolli and Roy).

The global pace of artificial intelligence (AI) advancement has rapidly accelerated in recent times. Meanwhile, AI approaches are also excellent tools for investigating the gut microbiome and drug metabolisms. In the present topic, Malwe and Sharma provided a comprehensive summary detailing the extensive metabolic capabilities of the gut microbiome on xenobiotic molecules. They further expanded on how AI and machine learning can be utilized to predict the specific gut microbiome and its enzymes engaged in the biotransformation processes of xenobiotics (Malwe and Sharma). Moreover, in their pursuit of exploring the gut microbiome's role in advancing personalized medicine, Huang et al. offered a comprehensive overview detailing how the gut microbiome boosts drug efficacy. They also delved into the specific mechanisms through which the gut microbiome deactivates certain medications. By compiling and reviewing the current understanding on the interaction between the gut microbiome and drugs, the authors have highlighted the gut microbiome as a novel key player to consider in personalized medicine (Huang et al.). Furthermore, Tan et al. proposed a new prediction model called MDSVDNV, which employed the Node2vec network embedding approach alongside the singular value decomposition (SVD) matrix decomposition method to predict microbe–drug interactions. Based on the successful feature extraction and learning of the microbe–drug network, the performance of the model was evaluated and was found to outperform the other existing competing models, as demonstrated by the test results (Tan et al.). This endeavor offers promising methods for uncovering latent microbe–drug interactions in the future.

Collectively, this topic presents significant scientific evidence elucidating the relationship between gut microbiota and drug metabolism/efficacy. These studies will accelerate our understanding of the molecular mechanisms involved in the gut microbiome and drug metabolism. We anticipate that these findings will aid in potential therapeutic targets for drug development and personalized medicine.

## Author contributions

J-HW: Writing—original draft, Writing—review & editing. D-WL: Writing—review & editing. MY: Writing—review & editing. XZ: Writing—review & editing.
